# Supplementary material for: CryoDRGN-AI: Neural ab initio reconstruction of challenging cryo-EM and cryo-ET datasets
Source: bioRxiv. 2025 Apr 28:2024.05.30.596729. Originally published 2024 Jun 2. Preprint. [Version 3] doi: 10.1101/2024.05.30.596729 (PMC11160740; doi:10.1101/2024.05.30.596729)
Supplement: 4 [file NIHPP2024.05.30.596729v3-supplement-4.pdf]

bioRxiv preprint doi: https://doi.org/10.1101/2024.05.30.596729; this version posted April 28, 2025. The copyright holder for this preprint (which was not certified by peer review) is the author/funder, who has granted bioRxiv a license to display the preprint in perpetuity. It is made available under aCC-BY 4.0 International license.

Supplementary Tables

Dataset	EMPIAR	SPA/ET	D	Å/pix.	Particles	Tilts	z_{dim}	Epochs	Time	GPUs
Pre-catalytic spliceosome [17]	10180	SPA	128	4.25	327,490	–	4	100	04:41 h	4xA100
Assembling ribosome [16]	10076	SPA	256	1.64	131,899	–	8	104	11:36 h	4xA100
Spike protein [18]	N/A	SPA	128	3.28	369,429	–	4	100	05:19 h	4xA100
DSL1/SNARE (run 1) [19]	11846	SPA	128	3.47	214,511	–	4	100	03:43 h	4xA100
DSL1/SNARE (run 2) [19]	11846	SPA	128	3.47	75,854	–	4	100	02:11 h	4xA100
V-ATPase (run 1) [20]	10874	SPA	128	3.97	267,216	–	4	101	08:40 h	4xA100
V-ATPase (run 2) [20]	10874	SPA	128	3.97	177,481	–	4	102	07:05 h	4xA100
Ankyrin [21]	11043	SPA	128	2.92	710,437	–	4	720	48:04 h	4xA100
70S Ribosome [22]	10499	ET	128	3.90	18,466	11	16	108	46:02 h	4xA100
Synthetic 80S ribosome [77]	N/A	SPA	128	3.77	100,000	–	–	30	01:25 h	4xA100
Synthetic 1D rotation [5]	N/A	SPA	128	6.00	50,000	–	8	100	03:02 h	4xA100

Table 1: **Datasets and cryoDRGN-AI training settings.** D refers to the box size in pixels.

Dataset	EMPIAR	Orig. box size	Orig. pixel size (Å)	Orig. number of particles	Best EMDB resolution (Å)
Pre-catalytic spliceosome [17]	10180	320	1.699	327,490	3.6
Assembling ribosome [16]	10076	320	1.31	131,899	3.7
DSL1/SNARE [19]	11846	400	1.114	469,193	4.5
Ankyrin [21]	11043	450	0.83	710,437	2.4

Table 2: **Original parameters of the datasets processed from picked particles deposited on EMPIAR.**

bioRxiv preprint doi: https://doi.org/10.1101/2024.05.30.596729; this version posted April 28, 2025. The copyright holder for this preprint (which was not certified by peer review) is the author/funder, who has granted bioRxiv a license to display the preprint in perpetuity. It is made available under aCC-BY 4.0 International license.

Supplementary Figures

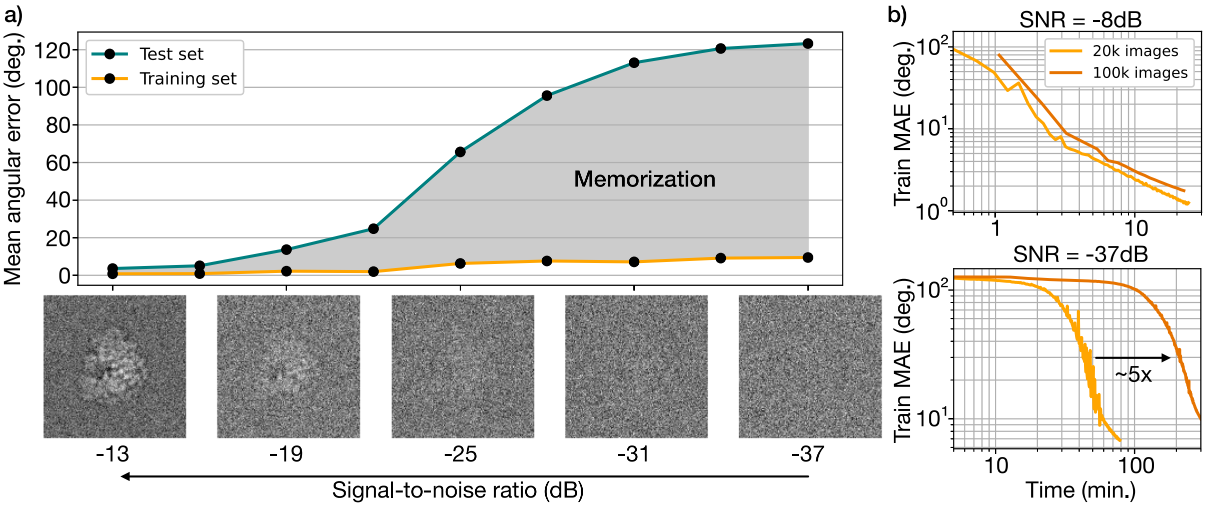

Supplementary Figure S1: **Memorization issue on high-noise datasets. a)** Mean angular error of a neural-based pose predictor trained on increasingly noisy synthetic datasets. While the training-time error is stable, the gap with the test-time error increases with noise. **b)** Training-time mean angular error (MAE) as a function of time on small (20k images) and large (100k images) datasets. On low-noise datasets (-8 dB), convergence time does not depend on the dataset size, On high-noise datasets (-37 dB), convergence time increases with the dataset size. This "memorization cost" invalidates the benefits of the amortized approach on high-noise datasets. See Methods for experimental details.

bioRxiv preprint doi: https://doi.org/10.1101/2024.05.30.596729; this version posted April 28, 2025. The copyright holder for this preprint (which was not certified by peer review) is the author/funder, who has granted bioRxiv a license to display the preprint in perpetuity. It is made available under aCC-BY 4.0 International license.

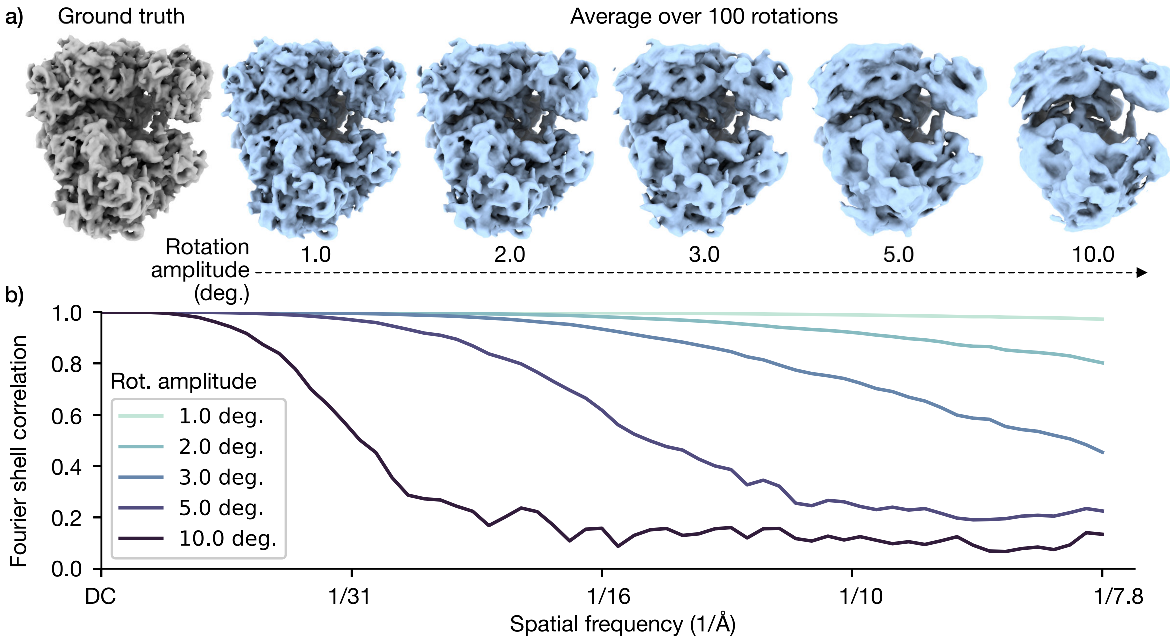

Supplementary Figure S2: **Influence of pose error on resolution.** To illustrate the impact of inaccurate poses on the reconstructed density map, 100 randomly rotated density maps of the 80S ribosome (128x128x128, 3.77 Å/pix.) are averaged, using a rotation amplitude between 1 and 10 degrees. **a)** Ground truth and averaged density maps for different rotation amplitudes. **b)** Fourier shell correlation for different rotation amplitudes.

bioRxiv preprint doi: https://doi.org/10.1101/2024.05.30.596729; this version posted April 28, 2025. The copyright holder for this preprint (which was not certified by peer review) is the author/funder, who has granted bioRxiv a license to display the preprint in perpetuity. It is made available under aCC-BY 4.0 International license.

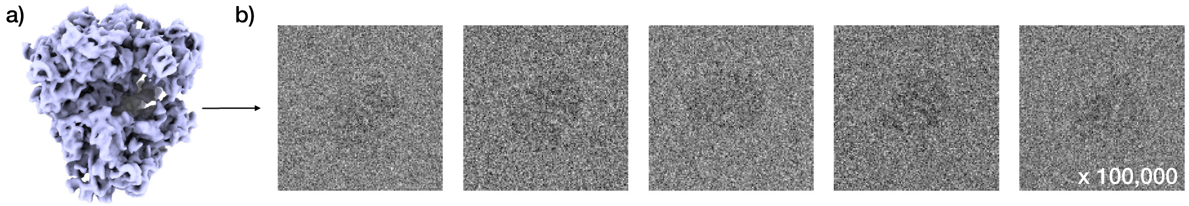

c)

Mean Error	CryoSPARC	CryoSPARC (refined)	HPS only	**Cryo-DRGN-AI**
Out-of-plane (deg.)	3.81	0.64	0.92	**0.62**
In-plane (deg.)	3.67	0.57	0.81	**0.56**
Translation (pix.)	0.32	**0.13**	0.17	0.14
Resolution (pix.)	2.4	**2.1**	2.4	2.2

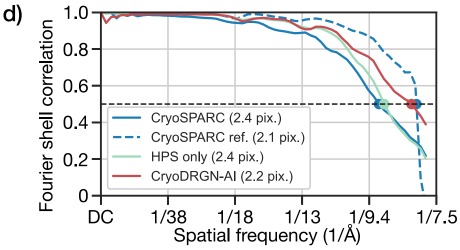

Supplementary Figure S3: **Homogeneous reconstruction of synthetic datasets.** **a)** Ground truth density map of the 80S ribosome. **b)** Five example particle images (100,000 particles, 128x128, 3.77 Å/pix). **c)** Pose error for cryoDRGN-AI, cryoDRGN-AI using HPS only and cryoSPARC [15]. **d)** FSC curves between the reconstructed density map and the ground truth density map (resolution at FSC 0.5 between parenthesis).

bioRxiv preprint doi: https://doi.org/10.1101/2024.05.30.596729; this version posted April 28, 2025. The copyright holder for this preprint (which was not certified by peer review) is the author/funder, who has granted bioRxiv a license to display the preprint in perpetuity. It is made available under aCC-BY 4.0 International license.

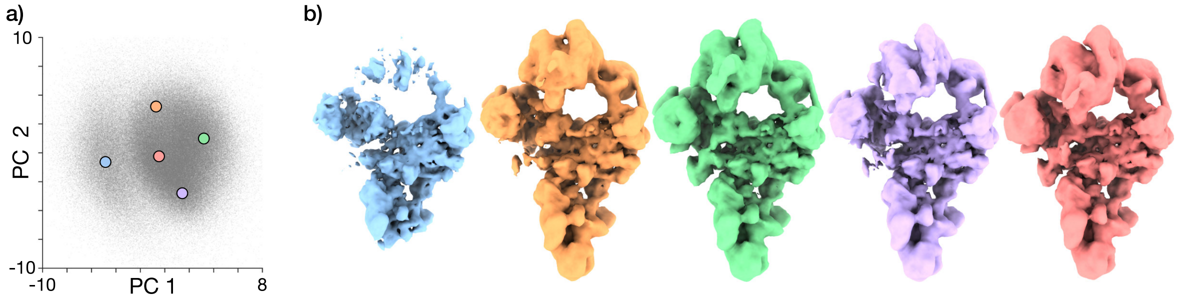

Supplementary Figure S4: **Additional visualizations of the latent space for the spliceosome dataset. a)** UMAP visualization of the latent embeddings with 5 centroids obtained by k-means clustering. **b)** Associated density maps.

bioRxiv preprint doi: https://doi.org/10.1101/2024.05.30.596729; this version posted April 28, 2025. The copyright holder for this preprint (which was not certified by peer review) is the author/funder, who has granted bioRxiv a license to display the preprint in perpetuity. It is made available under aCC-BY 4.0 International license.

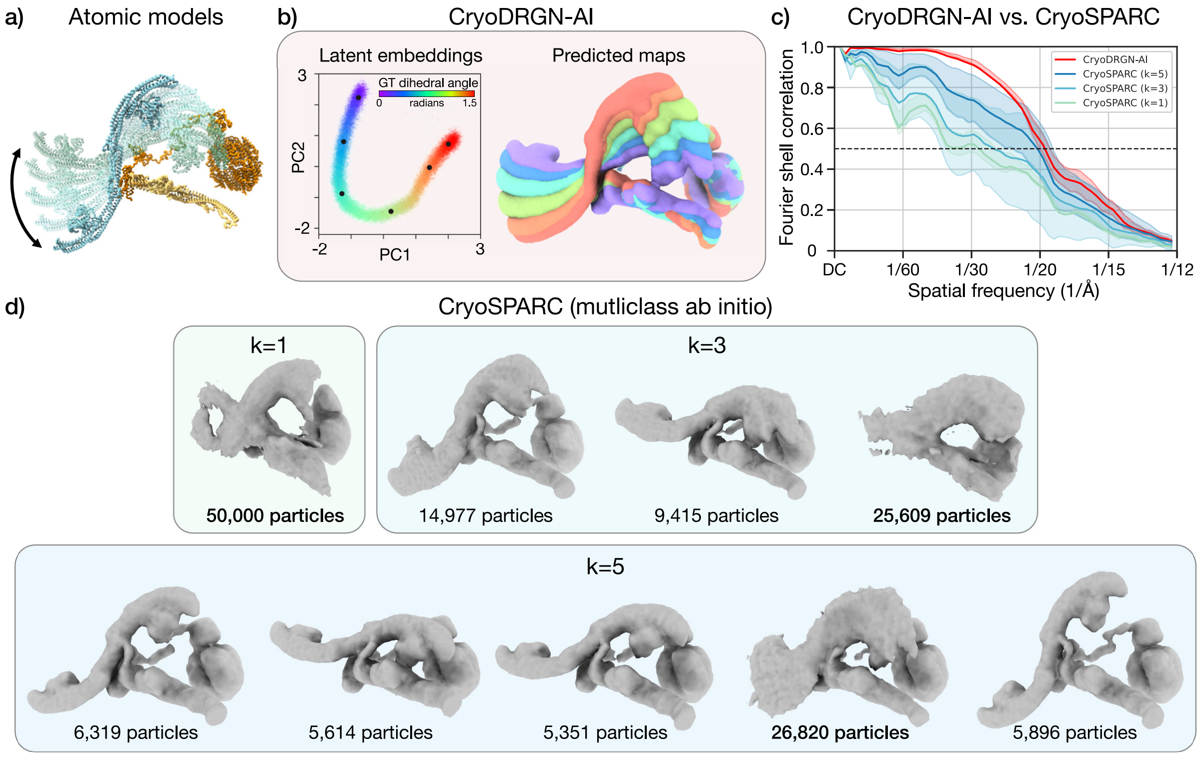

Supplementary Figure S5: **Results of cryoDRGN-AI on a synthetic dataset with strong conformational heterogeneity and comparison to cryoSPARC (multiclass *ab initio*). a)** The dataset simulates strong conformational heterogeneity (see Methods). We show 5 models separated by a 0.3 rad. increment rotation along a dihedral angle. **b)** Output of cryoDRGN-AI. PCA on the latent embeddings on the left (the hue represents, for each image, the true dihedral angle) and associated density maps on the right (sampled on black dots). **c)** Comparison between cryoDRGN-AI and cryoSPARC multiclass *ab initio*. We show the mean and inter-quartile range of the per-image FSC computed on 50 images uniformly sampling the range of motion. **d)** Results obtained with cryoSPARC multiclass *ab initio* (default parameters), using different numbers of classes. We indicate the number of particles associated to each class and bold the highest number.

bioRxiv preprint doi: https://doi.org/10.1101/2024.05.30.596729; this version posted April 28, 2025. The copyright holder for this preprint (which was not certified by peer review) is the author/funder, who has granted bioRxiv a license to display the preprint in perpetuity. It is made available under aCC-BY 4.0 International license.

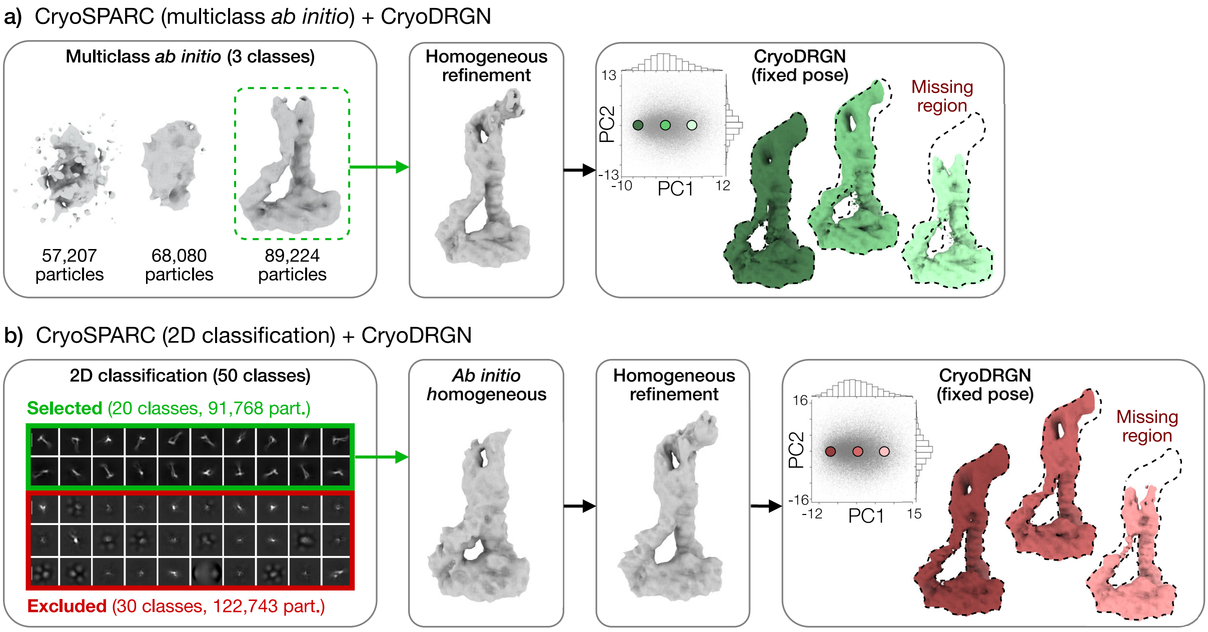

Supplementary Figure S6: **Comparison to other cryo-EM workflows for the DSL1 dataset, based on the cryoSPARC software [15]. a)** Spurious particles are filtered using multiclass ab initio reconstruction with 3 classes. Poses are then refined with a step of homogeneous refinement. The filtered and posed particle stack is finally processed with cryoDRGN [5]. **b)** Filtering is done by 2D classification and followed by a step of ab initio reconstruction and a step of homogeneous refinement. The posed particle stack is then processed with cryoDRGN.

bioRxiv preprint doi: https://doi.org/10.1101/2024.05.30.596729; this version posted April 28, 2025. The copyright holder for this preprint (which was not certified by peer review) is the author/funder, who has granted bioRxiv a license to display the preprint in perpetuity. It is made available under aCC-BY 4.0 International license.

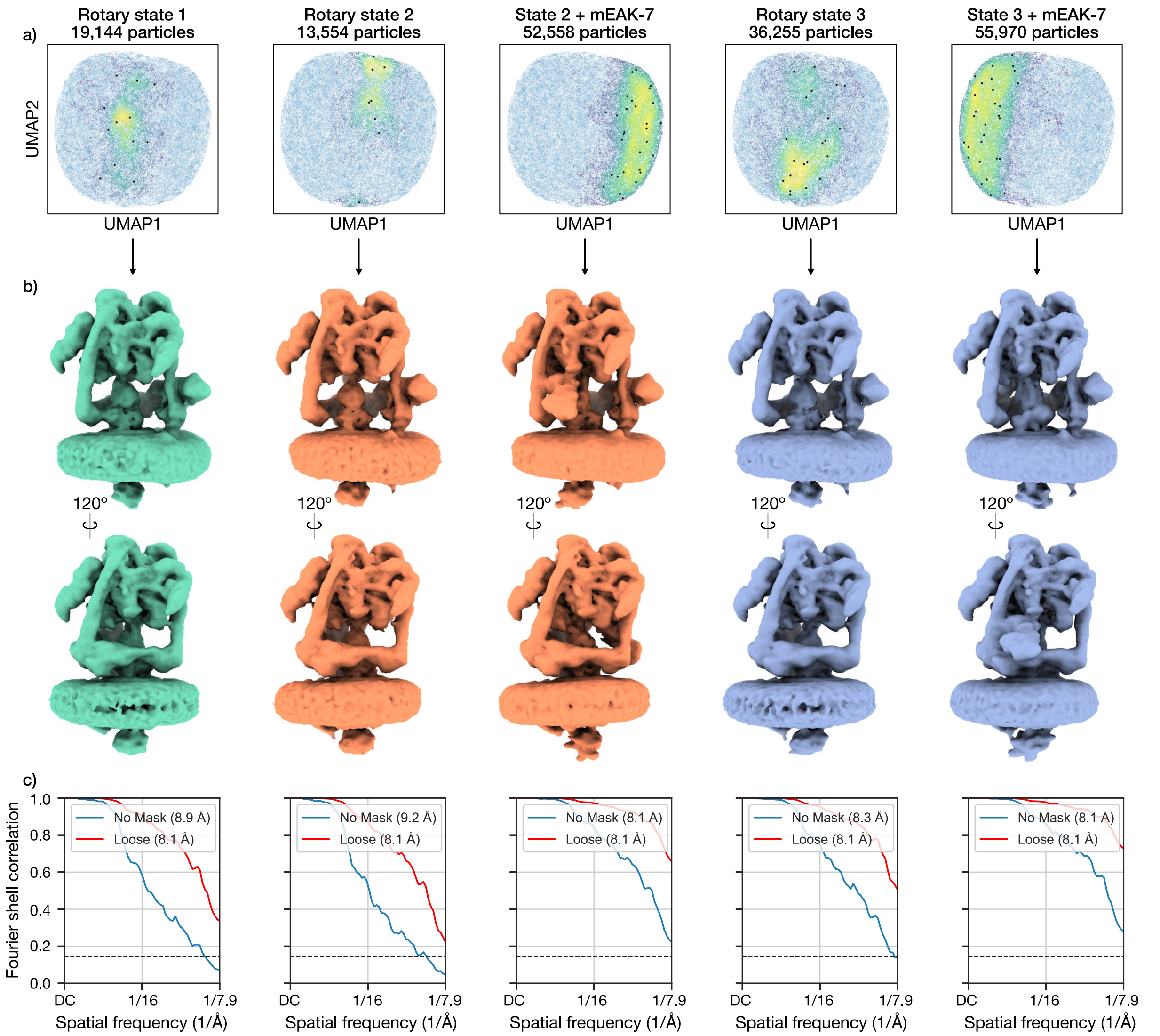

Supplementary Figure S7: **Backprojections of V-ATPase states. a)** UMAP visualizations of the latent embeddings, colored by the Gaussian kernel density estimate of the selected particles from each state. **b)** Two views of backprojected density maps from the selected particles of each state. **c)** Half-map FSC with soft mask at half of maximum value, 15 Åcosine edge width, and 25 Ådilation.

bioRxiv preprint doi: https://doi.org/10.1101/2024.05.30.596729; this version posted April 28, 2025. The copyright holder for this preprint (which was not certified by peer review) is the author/funder, who has granted bioRxiv a license to display the preprint in perpetuity. It is made available under aCC-BY 4.0 International license.

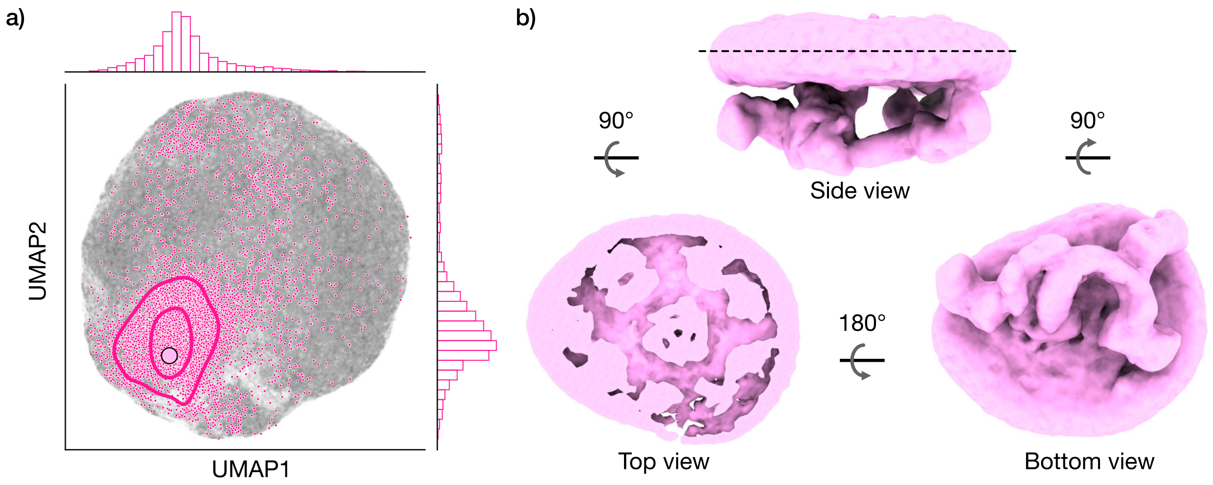

Supplementary Figure S8: **3D classification with cryoSPARC [15] on the ankyrin dataset, using the poses estimated by cryoDRGN-AI and 80 classes. a)** UMAP of the latent embeddings with cryoDRGN-AI (grey). The large pink circle indicates the conformation of the supercomplex shown in Figure 5. The smaller dots indicate the conformations of the particles belonging to the supercomplex class, according to cryoSPARC (8,523 particles). These conformations are approximated with a Gaussian KDE shown with pink contours. **b)** Three views of the supercomplex state reconstructed with cryoSPARC "3D classification", using cryoDRGN-AI-predicted poses (one class out of 80).

bioRxiv preprint doi: https://doi.org/10.1101/2024.05.30.596729; this version posted April 28, 2025. The copyright holder for this preprint (which was not certified by peer review) is the author/funder, who has granted bioRxiv a license to display the preprint in perpetuity. It is made available under aCC-BY 4.0 International license.

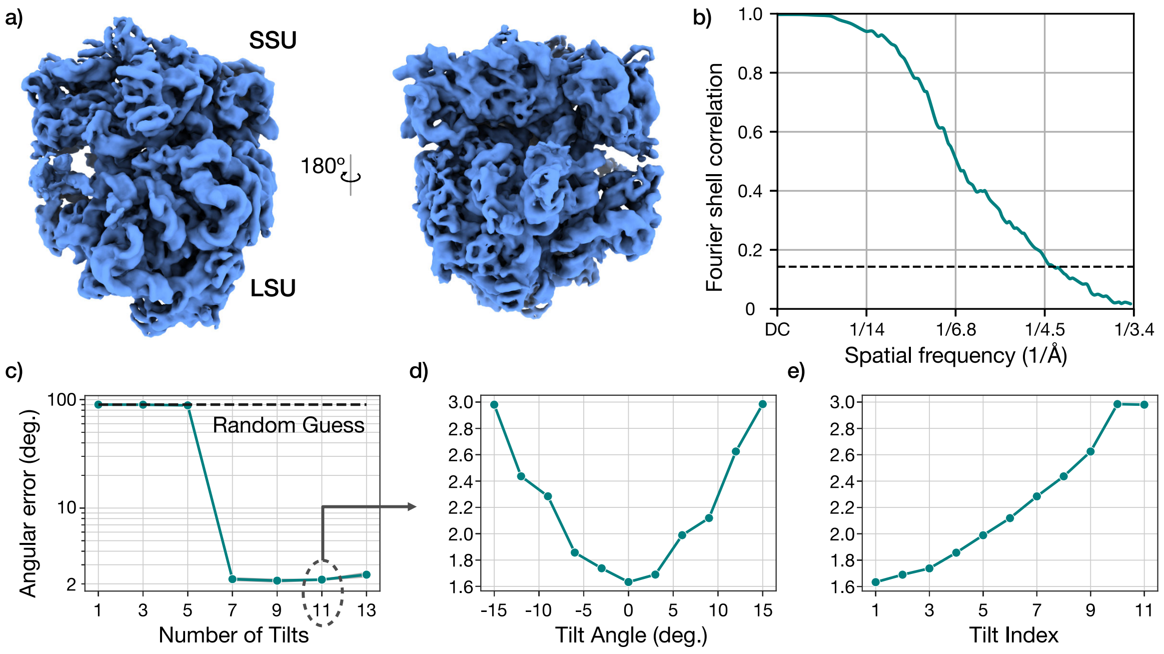

Supplementary Figure S9: **Homogeneous reconstruction of the "A, P stat" and pose accuracy of subtomogram reconstruction on the mycoplasma pneumoniae 70S ribosome.** **a)** Reconstructed density map from 14,857 selected particles from Rangan et al. [12] (11 tilts per particle, 163,427 subtilt images total, 294x294, 1.70 Å/pix.). **b)** Half-map FSC with soft mask at half of maximum value, 15 Åcosine edge width, and 25 Ådilation. **c, d, e)** Mean out-of-plane angular error vs. number of tilts used for reconstruction (c), vs. tilt angle (d) and vs. tilt index (e). d and e were obtained using 11 tilts per particle.

bioRxiv preprint doi: https://doi.org/10.1101/2024.05.30.596729; this version posted April 28, 2025. The copyright holder for this preprint (which was not certified by peer review) is the author/funder, who has granted bioRxiv a license to display the preprint in perpetuity. It is made available under aCC-BY 4.0 International license.

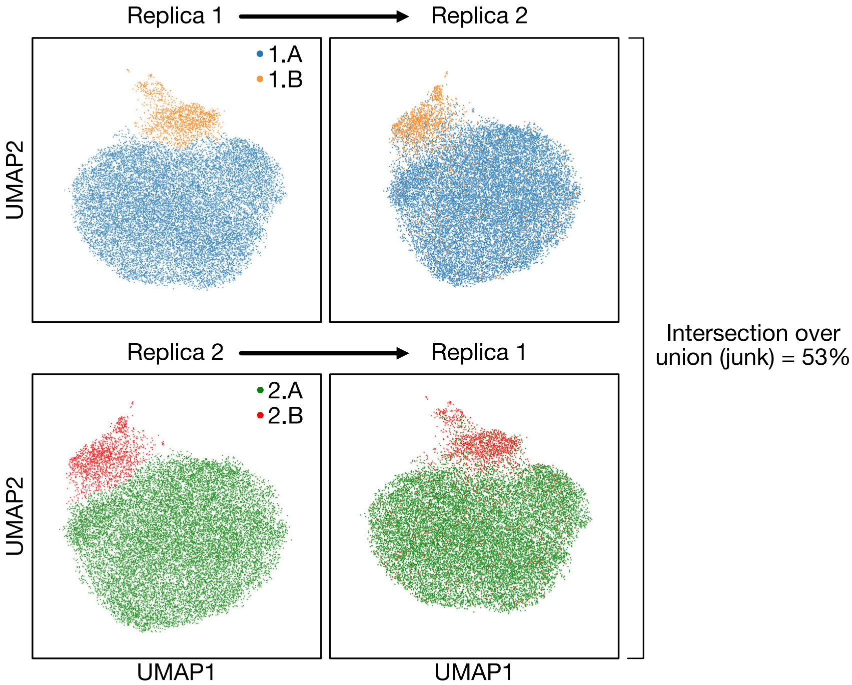

Supplementary Figure S10: **Consistent classification of junk in the mycoplasma pneumoniae 70S ribosome dataset.** We run two independent experiments (replica 1 and 2) and manually select an outlying cluster in the UMAP plots (1.B and 2.B). Each particle is labeled according to the cluster it appears in after reconstruction 1 (resp. 2), and their distributions are shown in the UMAP plot of reconstruction 2 (resp. 1). Intersection over union would be 4.8% if the two classifications were uncorrelated.
